# Establishment of an interleukin-1β-induced inflammation-activated endothelial cell-smooth muscle cell-mononuclear cell co-culture model and evaluation of the anti-inflammatory effects of tanshinone IIA on atherosclerosis

**DOI:** 10.3892/mmr.2015.3668

**Published:** 2015-04-23

**Authors:** YUJIE LI, YAN GUO, YING CHEN, YAJIE WANG, YUN YOU, QING YANG, XIAOGANG WENG, QI LI, XIAOXIN ZHU, BINGBING ZHOU, XUCEN LIU, ZAIPENG GONG, RUIJIE ZHANG

**Affiliations:** Department of Pharmacokinetics of Chinese Materia Medica, Institute of Chinese Materia Medica, China Academy of Chinese Medical Sciences, Beijing 100700, P.R. China

**Keywords:** atherosclerosis, inflammation, co-culture, endothelium, smooth muscle cell, monocyte, interleukin-1

## Abstract

Increasing evidence supports the hypothesis that inflammatory reactions serves an important function in the formation, progression and plaque rupture of atherosclerosis. Interleukin (IL)-1 primarily induces inflammation and is closely associated with the inflammatory environment and the formation of atherosclerosis. The present study aimed to establish an *in vitro* model for the evaluation of drug efficacy in the intervention of atherosclerosis from the inflammatory perspective, and to observe the anti-inflammatory effects of tanshinone IIA and andrographolide on atherosclerosis. The IL-1β-induced inflammation-activated endothelial cell (EC)-smooth muscle cell (SMC)-mononuclear cell (MC) co-culture model was established, based on the changes in a series of atherosclerosis-associated inflammatory markers secreted by ECs and SMCs. The expression of connexin in ECs, adhesion of MCs and changes in inflammatory signalling molecules were selected as evaluation indices for the inflammatory microenvironment of atherosclerosis. The use of this model revealed that tanshinone IIA exhibited significant efficacy against atherosclerosis and its inflammatory reactions. Inflammatory reactions were regarded as the primary mechanism underlying atherosclerosis. The established model simulated a series of relevant changes in the arterial wall under the inflammatory cytokines with oxidized low-density lipoprotein during the atherosclerotic process. The present study presented a reliable method for the identification of drugs with potential anti-inflammatory activity in atherosclerosis, for investigating the mechanisms of action, considering the improvement of the inflammatory state and the increase in plaque stability observed.

## Introduction

Increasing evidence supports the hypothesis that inflammation serves a function in the initiation, progression and plaque rupture of the acute coronary syndrome, atherosclerosis (1,2). A number of inflammatory biomarkers have been identified (3), thereby facilitating the development of several novel therapeutic targets and intervention methods for atherosclerosis by targeting inflammation and immune-associated factors (4). The Canakinumab Anti-inflammatory Thrombosis Outcome (5) and Cardiovascular Inflammation Reduction Trials (6), were initiated to obtain direct evidence on the reduction of cardiovascular risks through intervention of the inflammatory reaction. The results of these studies may initiate a new era for the treatment of coronary heart diseases (7).

The inflammatory reactions, mediated by the interactions between cells in the vascular wall and leukocytes, are involved in the progression of atherosclerosis, tumour development, allergic reactions and other diseases (8). The co-culture of endothelial cells (ECs) and smooth muscle cells (SMCs) is a frequently used method in investigations of atherosclerosis (9). EC-SMC co-culture enables the involvement of the growth factors, cytokines and other soluble mediators secreted by these two types of cells in intercellular communication (10–13). These mediators can mutually affect functions through receptor mediation, myoendothelial bridges between ECs and SMCs, establishment of gap junctions and changes in the extracellular matrix components (14–16). The EC, SMC and mononuclear cell (MC) co-culture system, established on a polyethylene microporous membrane has revealed that the direct contact between ECs and SMCs serves a key function in MC adhesion and infiltration, and also accelerates the adhesion dynamics of THP-1 cells with ECs (17). MC infiltration, foam cell formation, the expression of interleukin (IL)-8 in SMCs and collagen deposition have been observed in the EC-SMC co-culture system, using fibrin gel as the scaffold (18). In the EC-SMC-MC co-culture system, the advanced glycation endoproducts can upregulate the expression levels of IL-6, monocyte chemoattractant protein-1 (MCP-1) and other factors in the SMC (19). In these *in vitro* co-culture models, the primary focus was on MC adhesion, foam cell formation and associated aspects under oxidized low-density lipoprotein (ox-LDL) stimulation. Cellular inflammatory reactions are not the focus of these invetigations. Changes in ECs and MCs were examined, however minimal reference was made to corresponding changes in SMCs, particularly under inflammatory conditions.

Atherosclerosis is commonly understood as an inflammatory vascular disease, and targeting key inflammatory mediators through the inhibition of cytokine activities is a successful approach for preventing or slowing the progression of atherosclerosis (20). Previous studies have started to use inflammatory cytokines directly, as inflammatory inducers, to stimulate cells in the vascular wall to initiate the inflammatory process of atherosclerosis. This simulation may assist in evaluating drugs with potential anti-atherosclerotic and anti-inflammatory actions, with the most important stimulating factor being tumour necrosis factor (TNF)-α (20–22). In our previous study, we demonstrated that TNF-α exhibits a number of effects as a stimulator, however, TNF-α was not an ideal stimulator in this EC-SMC-MC model.

IL-1 is associated with the inflammatory environment, oxidative stress and the formation of atherosclerosis (7). An IL-1 gene-knockout (23) and the IL-1β monoclonal antibody (24) significantly reduce the formation of atherosclerotic plaques and suppress hypertension in mice (25). Cholesterol crystals can activate the NOD-like receptor family, pyrin domain containing 3 inflammasome in macrophages and stimulate the secretion of IL-1 and IL-1β, resulting in a chronic low-level inflammatory state, which increases the formation and progression of atherosclerosis (26). Certain previous studies have reported that six genes, including basic leucine zipper transcription factor, the BH3 interacting-domain death agonist apoptosis-associated gene, IL-1RN, complement receptor C3aR1, SEC61B and SLC43A3, are associated with the inflammation of atherosclerosis (27). IL-1 has been described as a promising novel target for future anti-athero-sclerotic drugs (25).

This present study hypothesized inflammatory reactions as the primary mechanism underlying atherosclerosis. IL-1β was used as the key stimulating factor, along with oxLDL, and was added into the EC-SMC-MC co-culture system. This set-up simulated atherosclerosis-induced changes in the cell functions and inflammatory microenvironment of three types of cell. This aimed to provide a novel method for the identification of drugs for use in atherosclerosis intervention from the inflammatory perspective, and to investigate the underlying mechanisms. The present study also investigated whether tanshinone IIA and andrographolide affected the early processes of atherosclerosis, including the inhibition of inflammatory markers, which are important for the EC-SMC-MC interaction and for plaque destabilization.

## Materials and methods

### EC monocultures and preparation of EC-SMC-MC co-cultures

Human umbilical vein smooth muscle cells (HUSMC; Sciencell Research Laboratories, Inc., Carlsbad, CA, USA) and human umbilical artery endothelial cells (HUAEC; Sciencell Research Laboratories, Inc.) were first incubated in separate culture dishes with smooth muscle conditioned medium and enriched culture medium (Sciencell Research Laboratories, Inc.), containing 5 (v/v) fetal calf serum (FCS; Gibco Life Technologies, Carlsbad, CA, USA), 100 IU/ml penicillin and 100 *µ*g/ml streptomycin (Beyotime Institute of Biotechnology, Jiangsu, China) at 37°C in a 5% CO_2_ and 95% air-humidified atmosphere. Cells between passages three and six were used to prepare the EC monocultures and EC-SMC co-cultures. On obtaining a sufficient number of HUAECs and HUASMCs (2–3×10^6^ cells), the cells were detached from the cell culture dish using trypsin-ethylene diamine tetra-acetic acid solution.

THP-1 cells (Sciencell Research Laboratories, Inc.) were cultured in 90% RPMI-1640 (Gibco Life Technologies), 10% FCS, 2 mM glutamine (Beyotime Institute of Biotechnology, Jiangsu, China), non-essential amino acids (Beyotime Institute of Biotechnology, Jiangsu, China), 1 mM sodium pyruvate (Beyotime Institute of Biotechnology, Jiangsu, China), 10 *µ*g/ml human insulin (Beyotime Institute of Biotechnology, Jiangsu, China) and 1 mM oxalacetate (Beyotime Institute of Biotechnology, Jiangsu, China) at 37°C and 5% CO_2_. The cells collected and adjusted to at density of 1×10^6^ cells/ml. The THP-1 cells were fluorescently labelled with 1 mmol/l 3′,6′-Di(O-acetyl)-4′, 5′-bis[N,N-bis(carboxymethyl)aminomethyl]fluorescein, tetraacetoxy-methyl ester (Calcein AM; Dojindo Molecular Technologies, Inc., Kumamoto, Japan) for 30 min at 37°C and 5% CO_2_. Following washing the cells with Hank’s Balanced Salt Solution (Zhongshan Golden Bridge Biotechnology Co., Ltd., Beijing, China), 1×10^5^ THP-1 cells/well were added to the upper chamber of the insert and incubated for 30 min at 37°C and 5% CO_2_.

To prepare an EC-SMC co-culture for use as a model of the arterial wall, Millicell insert units (PIHP01250; Millicell Millipore, Bedford, MA, USA) were placed in sterile tissue culture dishes and inverted, so that the outside of the membrane faced upward. The SMCs were seeded onto this outer membrane surface at a density of 1×10^5^ cells/cm^2^. The SMCs became adherent following 6 h of culture, and the insert units were placed in a 24-well culture plate in an upright position at 37°C and 5% CO_2_. The ECs were plated onto the inner surface of the membrane at a density of 1×10^5^ cells/well. These dishes were co-incubated for different durations at 37°C and 5% CO_2_ to form an EC-SMC co-culture system, according to the different requirements for the respective experiments, the EC-SMCs were co-cultured for 3–6 days). In addition, monocultures of ECs or SMCs were also prepared using the same method as the control. The supernatants from the upper and lower chambers of the insert were obtained, and the phosphatidylethanolamines (PE) membrane was cut to detect the markers and determine the EC-SMC co-culture condition.

The EC-SMC were co-cultured for 6 days and incubated with 100 *µ*g/ml oxLDL (Peking Union-Biology Co., Ltd., Beijing, China) or 100 *µ*g/ml oxLDL and 10 ng/ml IL-1β (PeproTech, Inc,. Rocky Hill, NJ, USA) for 4 h at 37°C and 5% CO_2_. The MCs were subsequently added into the upper chamber of the insert at a density of 1×10^5^ cells/cm^2^, and the mixture was co-cultured for another 20 h. The EC-SMCs were co-cultured for 6 days and the MCs were directly added to continue the co-culture for 20 h at 37°C and 5% CO_2_, which was used as a control to determine the inducer and sensitive marker of the inflammatory reaction in this model of atherosclerosis.

The EC-SMCs were co-cultured for 6 days and incubated with 100 *µ*g/ml oxLDL and 10 ng/ml IL-1β or different samples, including atorvastatin, indomethacin, tanshinone IIA and andrographolide, for 4 h at 37°C and 5% CO_2_. The MCs were subsequently added and co-cultured for another 20 h at 37°C and 5% CO_2_ to assess the effect of the drug on the inflammatory reaction in atherosclerosis.

### Enzyme-linked immunosorbent assay (ELISA)

The levels of TNF-α, MCP-1, ICAM-1, IL-10, endothelian 1 (ET-1) and nitric oxide (NO) released from the ECs, and the levels of IL-6, IL-8, matrix metalloproteinase (MMP)-2, MMP-9, transforming growth factor (TGF) β-1 and malondialdehyde (MDA) released from the SMCs were determined using a commercially available ELISA kit, according to the manufacturer’s instructions (RapidBio, West Hills, CA, USA). The co-culture incubation media (400–600 *µ*l) were collected following the removal of floating cells through centrifugation at 300 × g for 5 min at 4°C, using an Eppendorf 5424R centrifuge (Eppendorf, Hamburg, Gemany). The absorbance was measured at 450 nm using a multi-plate spectrophotometer (Bio-Tek Instruments, Inc., Winooski, VT, USA), according to the manufacturer’s instructions.

### Immunofluorescence staining and laser scanning confocal microscopy

The growth of cells in the inner PE membrane was assessed using an FV1000 laser scanning confocal microscope (Olympus, Tokyo, Japan). The PE membranes, which were cut from the insert, were washed three times with phosphate-buffered saline (PBS; Zhongshan Golden Bridge Biotechnology Co., Ltd.), fixed with 4% paraformaldehyde (Sigma-Aldrich) for 10 min and labelled with 1 mmol/l 3′-O-Acetyl-2′, 7′-bis(carboxyethyl)-4 or 5-carboxyfluorescein, diacetoxy-methyl ester (BCECF-AM; Dojindo Molecular Technologies, Inc.) for 10 min. Images were then captured and analysed using an FV1000 laser scanning confocal microscope.

The THP-1 cells were washed three times with PBS and the number of THP-1 cells bound to the ECs were measured by counting the number of adherent fluorescence-labelled THP-1 cells observed under a TE2000S fluorescence microscope (Nikon, Tokyo, Japan). This microscope encompassed a surface area of 0.314 mm^2^. A total of six areas were measured, with the results expressed as the number of THP-1 cells/mm^2^.

The expression of connexin-43 on the surface of the ECs was estimated by subtracting the mean fluorescence intensity of the cells labelled with the non-specific antibody from that of the connexin-43 antibody-labeled cells. All experiments were performed at least three times. Alexa Fluor 647-conjugated connexin-43 staining (Invitrogen Life Technologies, Carlsbad, CA, USA) was performed, according to the manufacturer’s instructions. Images were captured and analysed using an FV1000 laser scanning confocal microscope, equipped with an FV10-ASW viewer 2.0 image processing system (Olympus).

### RNA extraction and estimation of mRNA levels

The total RNA was isolated from agonist-stimulated or quiescent cells using TRIzol reagent (Invitrogen Life Technologies) and was reverse transcribed using a reverse transcription system (Promega, Madison, WI, USA), according to the manufacturer’s instructions (Invitrogen Life Technologies). Quantitative polymerase chain reaction (qPCR) was performed using a 7500 real-time PCR system (Applied Biosystems, Foster City, CA, USA) with SYBR Green PCR Master mix (Applied Biosystems), according to the manufacturer’s instructions. The mRNA expression levels of nuclear factor (NF)-κB p65 and peroxisome proliferator-activated receptor (PPAR)γ were normalized against that of glyceraldehyde-3-phosphate dehydrogenase (GAPDH), and were subsequently quantified. The following sequences of the forward and reverse primer pairs were used: NF-κB p65, forward 5′-GTTCACAGACCTGGCATCCGT-3′ and reverse 5′-AGAAGTCCATGTCCGCAATG-3′; PPARγ, forward 5′-ATGCTTGTGAAGGATGCAA G-3′ and reverse 5′-GATGGCATTATGAGACATCCC-3′ and GAPDH, forward 5′-ACCACAGTCCATGCCATCAC-3′ and reverse 5′-TCCACCAC CCTGTT G CTGTA-3′. All primers were synthesized by Taihe Biotechnology Co., Ltd., (Beijing, China). The following conditions were used: Denaturation at 95°C for 2 min; 45 cycles of 95°C for 20 sec, 58°C for 25 sec and 72°C for 30 sec, with a final fluorescence measurement. The data were normalized against the control and fold-changes were calculated using the 2^−∆∆Ct^ method. All reactions were performed in triplicate, using samples derived from three independent experiments.

### Statistical analysis

The data are expressed as the mean ± standard deviation. The experimental groups were compared by one-way analysis of variance. P<0.05 was considered to indicate a statistically significant difference.

## Results

### Cell growth and expression levels of inflammatory markers in EC or SMC monoculture and EC-SMC co-culture

**Figure 1:**
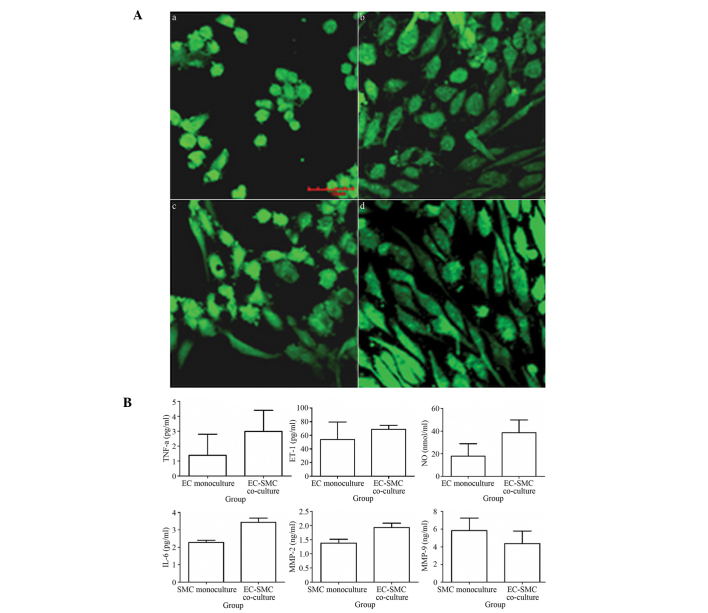
Comparison between EC and SMC monoculture and EC-SMC co-culture following culture for 3 days. (A) Fluorescence microscopy images of cell growth (BCECF-labeled; scale bar=50 *µ*m). (a) EC monoculture, (b) EC in co-culture, (c) SMC monoculture and (d) SMC in co-culture. (B) Concentrations of the atherosclerosis-associated inflammatory markers in the supernatant of the ECs and SMCs. The data are expressed as the mean ± standard deviation of six samples for each condition from a representative experiment (^*^P<0.05; ^**^P<0.01; ^***^P<0.001). EC, endothelial cell; SMC, smooth muscle cell; TNF, tumour necrosis factor; ET, endothelian; NO, nitric oxide; IL, interleukin; MMP, matrix metalloproteinase.

To systematically investigate the changing patterns of inflammation-associated factors during the co-culture of cells in the vascular system, the cell growth and the expression levels of inflammatory markers were compared in the EC or SMC monoculture and co-culture for 3 days. The results demonstrated that the cells grew significantly faster in the EC-SMC co-culture system compared with the EC and SMC monoculture (Fig. 1A and B). The levels of TNF-α and NO in the EC supernatant (Fig. 1B), and IL-6 and MMP2 in the SMC supernatant (Fig. 1D) were significantly higher in the co-culture system compared with the EC or SMC monoculture. However, these differences were not statistically significant.

### Cell growth and the level of atherosclerosis-associated inflammatory markers in cell supernatant in EC-SMC co-culture for different durations

**Figure 2:**
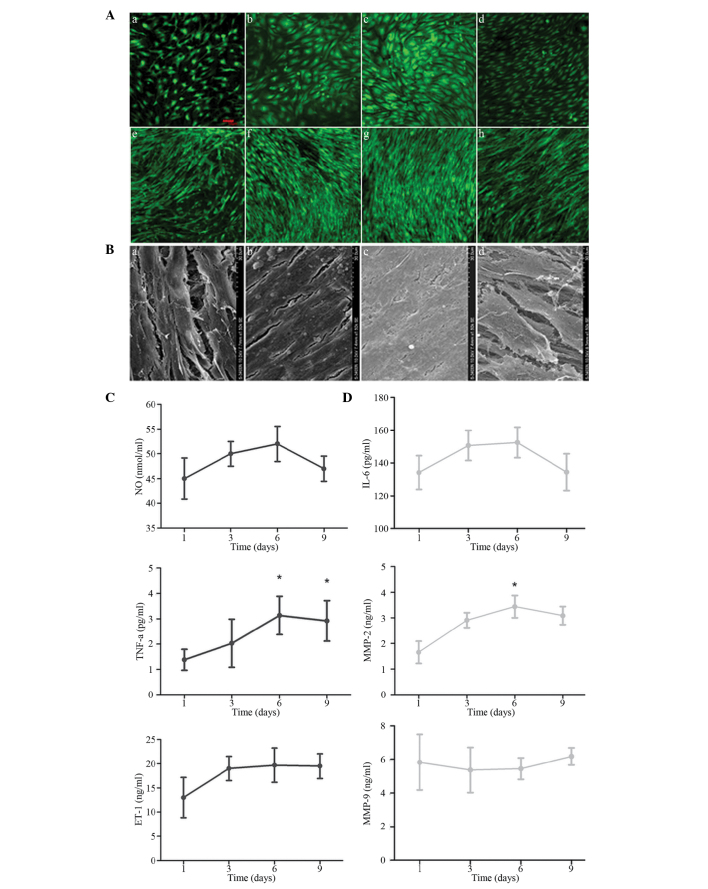
Comparison of the cell growth conditions and atherosclerosis-associated inflammatory markers in the cell supernatant at different time-points of EC-SMC co-culture. (A) Confocal laser scanning microscopy results. (a and e) EC and SMC growth conditions following EC-SMC co-culture for 3 days; (c and f) EC and SMC growth conditions following co-culture for 6 days; (c and g) EC and SMC growth conditions following co-culture for 9 days. (d and h) EC and SMC growth conditions following co-culture for 12 days, respectively (magnification, ×100; scale bar=50 *µ*m). (B) Electron microscopy of the SMC growth conditions following co-culture for 3, 6, 9 and 12 days (magnification, ×1,500). The concentrations of atherosclerosis-associated inflammatory markers in the cell supernatant of the (C) ECs and (D) SMCs following EC-SMC co-culture for 1–9 days, determined using ELISA. The data are expressed as the mean ± standard deviation (^*^P<0.05, vs. 1 day). EC, endothelial cell; SMC, smooth muscle cell; TNF, tumour necrosis factor; ET, endothelian; NO, nitric oxide; IL, interleukin; MMP, matrix metalloproteinase.

To understand the changing patterns of atherosclerosis-associated inflammatory markers at different time-points of EC-SMC co-culture, confocal microscopy (FV1000; Olympus; BCECF-labeled; excitation, 488 nm and emmission, 525 nm) and electron microscopy (Hitcahi S-3400N scanning electron microscope; were used to observe the growth of the ECs and SMCs following EC-SMC co-culture for 3, 6, 9 and 12 days. Based on these observations, the changes in atherosclerosis-associated inflammatory markers in the EC and SMC cell culture supernatants were further determined following EC-SMC co-culture for 1, 3, 6 and 9 days. The results demonstrated that the cells grew more efficiently following the EC-SMC co-culture compared with thte EC or SMC monoculture between 3 and 9 days, EC formed a dense monolayer between 6 and 9 days, and the SMCs exhibited multilayer growth and formed a lamellar structure, which was detected by electron microscopy. Following co-culture for 12 days, the cell conditions deteriorated, demonstrating significant exfoliation and widened intercellular space (Fig. 2A). Following 1 day of co-culture, the levels of ET-1, NO and TNF-α in the EC cell supernatant increased between 3 and 9 days. In addition, the increased levels of TNF-α observed at 6 and 9 days was statistically significant (Fig. 2B). The levels of MMP2 and IL-6 in the SMC supernatant exhibited an increasing trend between 3 and 9 days in the co-culture system, and the increase in MMP2 after 6 days was statistically significant (Fig. 2C). Based on the comprehensive analysis of the results, the subsequent experiments were performed following EC-SMC co-culture for 6 days.

### Level of atherosclerosis-associated inflammatory markers in the IL-1β-induced inflammation-activated EC-SMC-MC co-culture model

**Figure 3:**
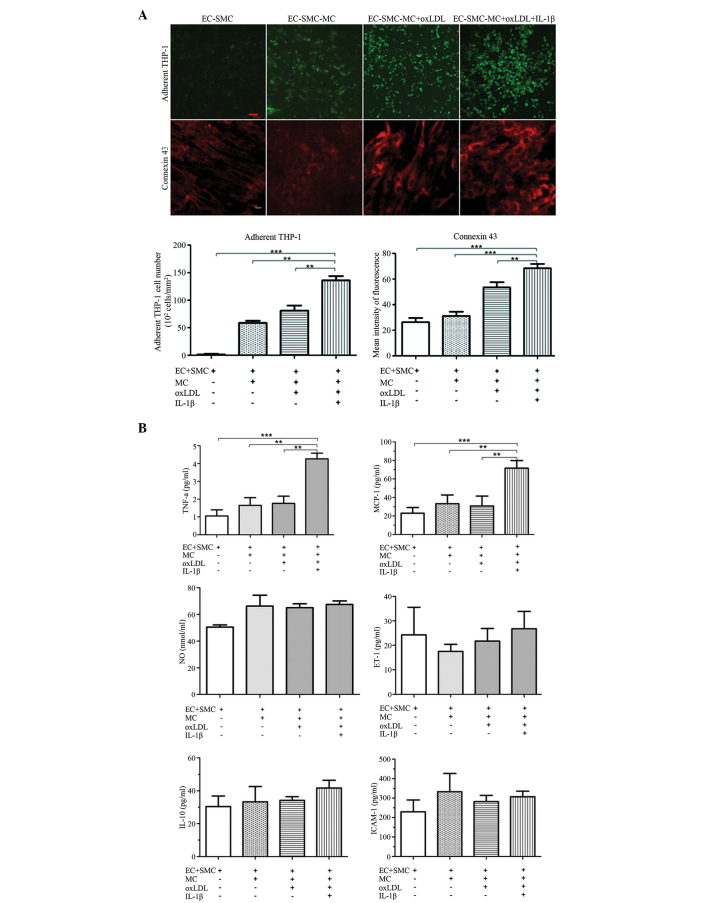

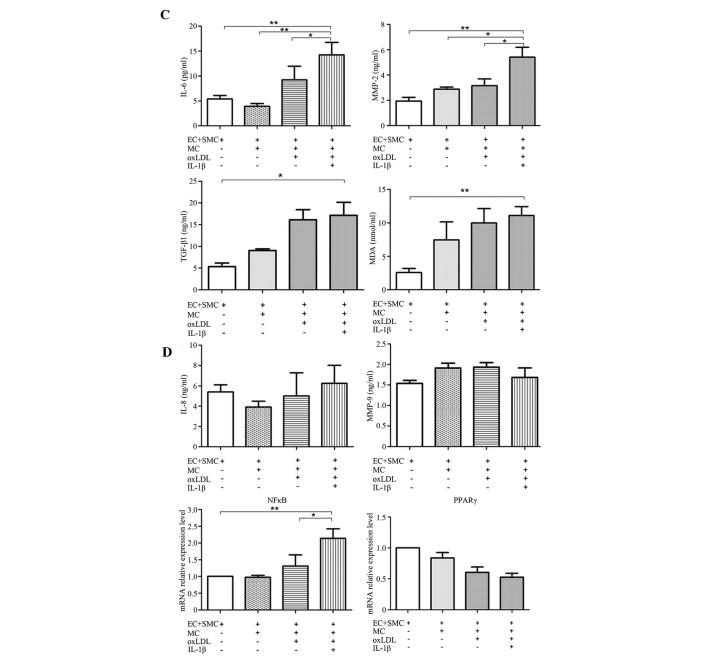
Comparison of the expression levels of atherosclerosis-associated inflammatory markers in EC-SMC co-culture, with or without MCs, oxLDL and IL-1β. (A) Confocal laser scanning microscopy images of EC-surface adhered MCs (scale bar=50 *µ*m) and the expression of connexin 43 (scale bar=10 *µ*m). (B) Concentrations of atherosclerosis-associated inflammatory markers in the EC supernatant in EC-SMC co-culture, with or without MCs, oxLDL and IL-1βs. The data are expressed as the mean ± standard deviation (^**^P<0.01; ^***^P<0.001, vs. EC-SMC-MC co-culture with oxLDL and IL-1β groups). EC, endothelial cell; SMC, smooth muscle cell; MC, monocyte; TNF, tumour necrosis factor; ET, endothelian; NO, nitric oxide; IL, interleukin; MMP, matrix metalloproteinase; MCP, monocyte chemoattractant protein; ICAM, intercellular adhesion molecule; TGF, transforming growth factor; oxLDL, oxidative low density lipoprotein; NF, nuclear factor; PPAR, peroxisome proliferator-activated receptor. (C) Expression levels of atherosclerosis-associated inflammatory markers in the SMC supernatant, with or without MCs, oxLDL and IL-1β, measured by enzyme-linked immunosorbent assay. (D) mRNA expression levels of NF-κB and PPARγ in the cells of EC layer were subjected to reverse transcription-quantitative polymerase chain reaction. The data are expressed as the mean ± standard deviation (^*^P<0.05; ^**^P<0.01, vs. EC-SMC-MC co-culture with oxLDL and IL-1β). EC, endothelial cell; SMC, smooth muscle cell; MC, monocyte; TNF, tumour necrosis factor; ET, endothelian; NO, nitric oxide; IL, interleukin; MMP, matrix metalloproteinase; MCP, monocyte chemoattractant protein; ICAM, intercellular adhesion molecule; TGF, transforming growth factor; oxLDL, oxidative low density lipoprotein; NF, nuclear factor; PPAR, peroxisome proliferator-activated receptor.

To simulate the inflammatory reaction process of the cells in the vascular wall during atherosclerosis, a series of atherosclerosis-associated reactions and the expression levels of inflammatory markers were compared. These markers included the EC-surface adherent MC counts, expression levels of EC-surface connexin-43, concentrations of TNF-α, MCP-1, ET-1, NO, ICAM-1 and IL-10 in the EC supernatant, concentrations of IL-6, IL-8, MMP-2, MMP-9, TGFβ-1 and MDA in the SMC supernatant, and the mRNA expression levels of NF-κB and PPARγ in the cells of the EC layer. This was performed under four culture conditions, including EC-SMC co-culture, EC-SMC-MC co-culture, MC co-culture with oxLDL-activated EC-SMC and MC co-culture with oxLDL and IL-1β-activated EC-SMC. The results demonstrated that the EC surface adherent MC count and the expression of EC-surface connexin-43 were significantly higher following co-culture of MC with oxLDL and IL-1β-activated EC-SMC (EC-SMC-MC+O+I group) compared with the other groups (Fig. 3A). The levels of TNF-α and MCP-1 in the EC supernatant (Fig. 3B); IL-6, MMP-2, TGFβ-1 and MDA in the SMC supernatant (Fig. 3C); and of NF-κB in the EC layer increased significantly in this group (Fig. 3D). Therefore, the conditions required for the establishment of this model were as follows: EC-SMC co-culture for 6 days, followed by incubation with 100 *µ*g/ml oxLDL and 10 ng/ml IL-1β for 4 h, followed by MC addition and co-culture for another 20 h.

### Validation of the IL-1β-induced inflammation-activated EC-SMC-MC co-culture model

**Figure 4:**
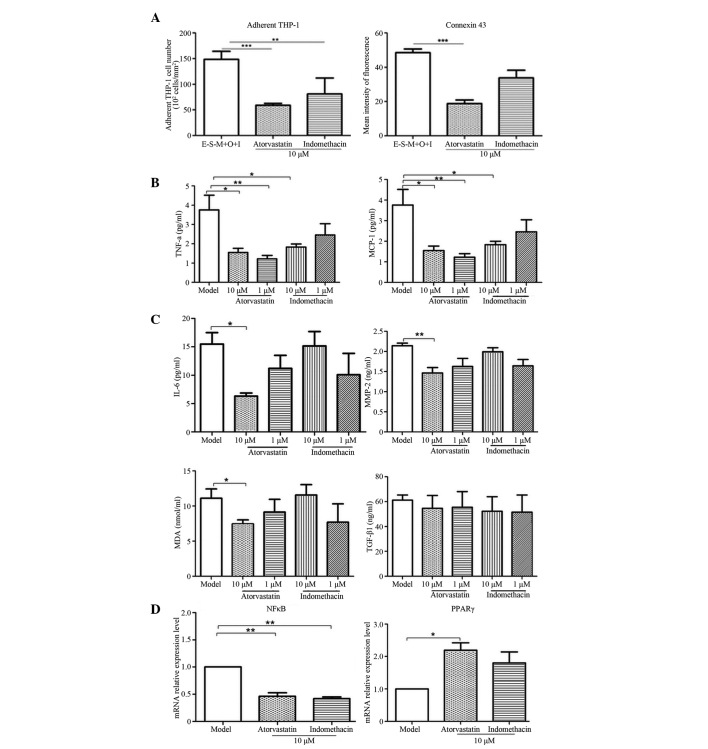
Effects of atorvastatin and indomethacin on atherosclerosis-associated inflammatory markers in the IL-1β-induced inflammation-activated EC-SMC-MC co-culture model. (A) EC-surface adhered MC count and the expression of Connexin 43. Concentrations of atherosclerosis-associated inflammatory markers in the (B) EC and (C) SMC supernatant were measured by ELISA. (D) The mRNA expression levels of NF-κB and PPARγ in cells from the EC layer were subjected to reverse transcription quantitative polymerase chain reaction. The data are expressed as the mean ± standard deviation (^*^P<0.05, ^**^P<0.01, ^***^P<0.001, vs. Model). E, endothelial cell; S, smooth muscle cell; M, monocyte; O, oxidated low density lipoprotein; I, interleukin-1β induced; TNF, tumour necrosis factor; IL, interleukin; MMP, matrix metalloproteinase; MCP, monocyte chemoattractant protein; TGF, transforming growth factor; NF, nuclear factor; PPAR, peroxisome proliferator-activated receptor.

To determine the reliability of the co-culture model, the model was validated using anti-atherosclerotic agent, atorvastatin, which has known lipid-lowering and anti-inflammatory effects, and the non-steroidal anti-inflammatory drug, indomethacin. The experimental results demonstrated that treatment with atorvastatin resulted in an effective reduction of the EC-surface adhered cell count and inhibition of the expression of connexin-43 (Fig. 4A), decreased levels of TNF-α and MCP-1 in the EC supernatant (Fig. 4B) and MMP-2, IL-6, and MDA in SMC supernatant (Fig. 4C), and downregulation of the mRNA expression of NF-κB and upregulation of the mRNA expression of PPARγ in the ECs (Fig. 4D). By contrast, indomethacin reduced the EC surface-adhered cell count, inhibited the secretion of TNF-α and MCP-1 by the ECs, and suppressed the mRNA expression of NF-κB. However, treatment with indomethacin revealed no significant effects on the other indices. These results suggested that this model effectively reflected the efficacy of anti-athero-sclerotic agents with an anti-inflammatory effect, and may be used to identify drugs with potential anti-inflammatory and anti-atherosclerotic activities, and to assess their efficacy.

### Evaluation of the anti-atherosclerotic effects of atorvastatin and indomethacin in the IL-1β-induced inflammation-activated EC-SMC-MC co-culture model

**Figure 5:**
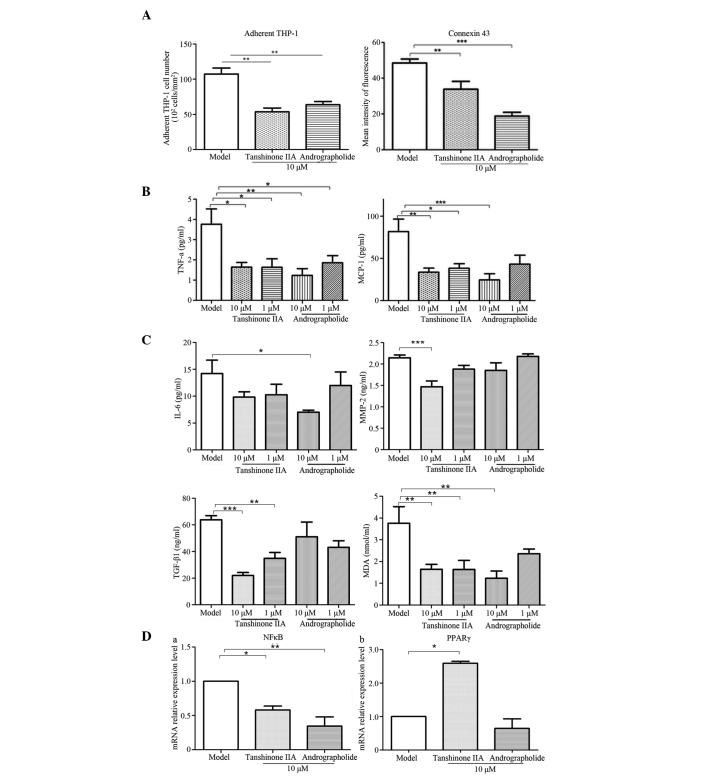
Effects of tanshinone IIA and andrographolide on atherosclerosis-associated inflammatory markers in the IL-1β-induced inflammation-activated EC-SMC-MC co-culture model. (A) EC-surface adhered MC count and the expression of Connexin 43. Concentrations of atherosclerosis-associated inflammatory markers in the (B) EC and (C) SMC supernatant were measured by ELISA. (D) The mRNA expression levels of NF-κB and PPARγ in cells from the EC layer were subjected to reverse transcription quantitative polymerase chain reaction. The data are expressed as the mean ± standard deviation (^*^P<0.05, ^**^P<0.01, ^***^P<0.001, vs. Model). EC, endothelial cell; SMC, smooth muscle cell; MC, monocyte; TNF, tumour necrosis factor; IL, interleukin; MMP, matrix metalloproteinase; MCP, monocyte chemoattractant protein; TGF, transforming growth factor; NF, nuclear factor; PPAR, peroxisome proliferator-activated receptor.

Based on the IL-1β-induced inflammation-activated co-culture model, the anti-athero-sclerotic efficacy of tanshinone IIA and andrographolide in exhibiting anti-inflammatory or anti-atheroscleroticactivity was comprehensively assessed. The present study investigated whether tanshinone IIA and andrographolide affected the early stages of atherosclerosis, including the inhibition of inflammatory markers. The results demonstrated that tanshinone IIA in the co-culture model inhibited the EC surface-adhered MC count and the expression of connexin-43, decreased the secretion of TNF-α and MCP-1 by the ECs, and TGFβ-1, MMP-2 and MDA in the SMC supernatant, and affected the mRNA expression levels of NF-κB and PPARγ in EC layer cells. Therefore, tanshinone IIA demonstrated significant anti-atherosclerotic and anti-inflammatory effects (Fig. 5). Andrographolide also inhibited certain indices in this model, which were principally associated with inflammation, demonstrating a significant anti-inflammatory effect, consistent with previous studies (28,29). The experimental results also suggested that tanshinone IIA affected the expression of inflammatory markers in atherosclerosis and may serve an important interventional function in the formation of atherosclerosis and plaque stabilization by inhibiting inflammatory reactions.

## Discussion

The interactions between ECs, SMCs and MCs contribute to the normal function of the vessel wall and to the pathogenesis of certain diseases, including atherosclerosis. Previous studies used the EC-SMC co-culture system in investigations of atherosclerosis (30–32). Takaku *et al* (31) observed the migration and differentiation of MCs and the formation of foam cells under oxLDL in the EC-SMC culture system in 1999, and subsequent studies have predominantly used oxLDL as a primary stimulant for the atherosclerotic process. EC-SMC co-culture with oxLDL can upregulate the expression levels of intercellular adhesion molecule (ICAM)-1, vascular cell adhesion protein-1 and other factors by ECs, and the release of TNF-α (33,34). Furthermore, the migratory function of ECs can vary, which variation is associated with the activation of histone deacety-lase 6 and downregulation of the expression of acetylated tubulin in ECs (35). In the EC-SMC co-culture system, SMCs can secrete vascular endothelial growth factor to stimulate the ECs to produce a series of changes, including cell proliferation, differentiation, migration and the deposition of extracellular matrix proteins (36). Co-cultured SMCs promote the adhesion of ECs by modulating the microtubule cytoskeleton polymerization state, which in turn activates the extracellular regulated kinase pathway and upregulates the expression of phosphorylated paxillin to accelerate focal adhesion formation (37). In the present study, to investigate the atherosclerosis-associated inflammatory reaction in the co-culture model, changes in a series of atherosclerosis inflammatory markers at different time-points and under different culture conditions were examined. The results demonstrated that the levels of TNF-α in the EC culture supernatant increased significantly following EC-SMC co-culture for 6 days (Fig. 2B), which was consistent with a previous report (35), and the levels of MMP-2 in the SMC culture supernatant increased significantly (Fig. 2C). Based on the overall evaluation of the cell growth conditions, EC-SMC co-culture for 6 days was determined as the suitable condition for subsequent experiments. In addition, differences in the expression of atherosclerosis-associated inflammatory factors under several co-culture conditions and stimulations were systematically observed and compared (Fig. 3). These results confirmed that the inflammatory changes were the most evident following co-culture of EC-SMC-MC combined with stimulation of ox-LDL and IL-1β. The extensive preliminary experiments enabled the screening of certain stable atherosclerosis-associated inflammation indicators, including the expression of the EC-surface connexin-43, the number of adherent MCs, changes in the series of inflammatory markers secreted by ECs and SMCs, and the changes in the inflammatory signalling molecules (Fig. 3).

Connexin-43 is a member of the connexin family, which forms intercellular gap junctions and is closely associated with inflammation. Connexin-43-knockdown alleviates the brain inflammation and glia activation induced by peripheral lipopolysaccharide injection (38). Connexin-43 is upregulated in the neointimal SMCs at an early stage of atherosclerosis in rabbits (39). The results of the present study demonstrated that connexin-43 is expressed in ECs for the entire duration of culture. This finding provides evidence of EC inflammatory stimulation and of ongoing intercellular communication between ECs and SMCs in the model, similar to previous studies (40,41), acting as an indicator of atherosclerosis-associated inflammation.

The atherosclerosis-associated factors in the SMC supernatant under inflammatory conditions were also observed. MMP-2 contributes to the development of atherosclerosis and, activated MMP-2 has been observed in human carotid endarterectomy specimens (42). A significant reduction in atherosclerotic plaques in the aortic sinus and arch were observed with the decrease in smooth muscle cell-positive area in MMP-2(−/−) in mice (43). The increased expression of MMP-2 was also a significant feature of the model in the present study. SMC proliferation and lipid peroxide product accumulation-associated indices, including TGFβ-1 and MDA, were also observed in the SMC supernatant, therefore this model can be used to evaluate the effects of drugs, focus-sing on pathological processes associated with atherosclerosis.

Danshen (DS) is a traditional Chinese medicine, which is commonly used for the treatment of cardiovascular and cerebrovascular diseases, and tanshinone is one of its active ingredients. Tanshinone IIA exhibits certain anti-atherosclerotic effects, protects cells from being injured by hydrogen peroxide (44), suppresses cholesterol accumulation and affects the formation of foam cell (45,46). Stumpf *et al* (46) reported that DS and its major ingredients significantly inhibited TNF-α-induced expression, the release of adhesion molecules, cytokines and chemokines, and the adenosine diphosphate-induced expression of platelet P-selectin. Andrographolide exhibits an anti-inflammatory effect by downregulating the p38 mitogen activated protein kinase, signal transducer and activator of transcription 3 and NF-κB pathways (47,48). The present study assessed whether tanshinone IIA and andrographolide exhibited the potential anti-inflammatory effects of atherosclerosis in the established experimental system. The results demonstrated that tanshi-none IIA exhibited significant efficacy against atherosclerosis and its inflammatory reactions. Tanshinone IIA inhibited the MMP-2 and NF-κB signalling pathways in the IL-1β-induced inflammation-activated co-culture model, as confirmed previously (49).

The IL-1β-induced inflammation-activated EC-SMC-MC co-culture model in the present study demonstrated that changes in the expression levels of a series of atherosclerosis inflammatory markers secreted by ECs, SMCs, levels of EC connexin, MC adhesion and changes in the inflammatory signalling molecules can be used as evaluation indices for the inflammatory microenvironment of atherosclerosis. This model was able to simulate a series of relevant changes in the cell structure and function of the arterial wall, character-ized by inflammatory reactions of inflammatory cytokines with oxLDL, a risk factor for atherosclerosis, during the atherosclerotic process. The establishment of this model system offers a reliable method for identifying drugs with potential anti-atherosclerotic activity, and for investigating the mechanisms of action to improve the inflammatory state and increase plaque stability. Based on this model, the present study revealed that tanshinone IIA affected the early stages of atherosclerosis through inflammatory reactions and plaque destabilization.

## References

[1] Libby P (2002). Inflammation in atherosclerosis. Nature.

[2] Wong BW, Meredith A, Lin D, McManus BM (2012). The biological role of inflammation in atherosclerosis. Can J Cardiol.

[3] Siegel D, Devaraj S, Mitra A, Raychaudhuri SP, Raychaudhuri SK, Jialal I (2013). Inflammation, atherosclerosis and psoriasis. Clin Rev Allergy Immunol.

[4] Charo IF, Taub R (2011). Anti-inflammatory therapeutics for the treatment of atherosclerosis. Nat Rev Drug Discov.

[5] Ridker PM, Thuren T, Zalewski A, Libby P (2011). Interleukin-1β inhibition and the prevention of recurrent cardiovascular events: rationale and design of the canakinumab anti-inflammatory thrombosis outcomes study (CANTOS). Am Heart J.

[6] Ridker PM (2009). Testing the inflammatory hypothesis of athero-thrombosis: scientific rationale for the cardiovascular inflammation reduction trial (CIRT). J Thromb Haemost.

[7] Verma S, Gupta M, Ridker PM (2012). Therapeutic targeting of inflammation in atherosclerosis: we are getting closer. Can J Cardiol.

[8] Libby P (2002). Inflammation in atherosclerosis. Nature.

[9] Davies PF (1986). Vascular cell interactions with special reference to the pathogenesis of atherosclerosis. Lab Invest.

[10] Campbell JH, Campbell GR (1986). Endothelial cell influences on vascular smooth muscle phenotype. Annu Rev Physiol.

[11] Powell RJ, Hydowski J, Frank O, Bhargava J, Sumpio BE (1997). Endothelial cell effect on smooth muscle cell collagen synthesis. J Surg Res.

[12] Powell RJ, Bhargava J, Basson MD, Sumpio BE (1998). Coculture conditions alter endothelial modulation of TGF-beta 1 activation and smooth muscle growth morphology. Am J Physiol.

[13] Lyle AN, Griendling KK (2006). Modulation of vascular smooth muscle signaling by reactive oxygen species. Physiology (Bethesda).

[14] Spagnoli LG, Villaschi S, Neri L, Palmieri G (1982). Gap junctions in myo-endothelial bridges of rabbit carotid arteries. Experientia.

[15] Fillinger MF, O’Connor SE, Wagner RJ, Cronenwett JL (1993). The effect of endothelial cell coculture on smooth muscle cell proliferation. J Vasc Surg.

[16] Powell RJ, Carruth JA, Basson MD, Bloodgood R, Sumpio BE (1996). Matrix-specific effect of endothelial control of smooth muscle cell migration. J Vasc Surg.

[17] Kinard F, Jaworski K, Sergent-Engelen T (2001). Smooth muscle cells influence monocyte response to LDL as well as their adhesion and transmigration in a coculture model of the arterial wall. J Vasc Res.

[18] Dorweiler B, Torzewski M, Dahm M (2006). A novel in vitro model for the study of plaque development in atherosclerosis. Thromb Haemost.

[19] Nam MH, Lee HS, Seomun Y, Lee Y, Lee KW (2011). Monocyte-endothelium-smooth muscle cell interaction in co-culture: proliferation and cytokine productions in response to advanced glycation end products. Biochim Biophys Acta.

[20] Desai A, Darland G, Bland JS, Tripp Ml, Konda VR (2012). META060 attenuates TNF-alpha-activated inf lam-mation, endothelial-monocyte interactions and matrix metalloproteinase-9 expression and inhibits NF-kappaB and AP-1 in THP-1 monocytes. Atherosclerosis.

[21] Moon MK, Lee YJ, Kim JS, Kang DG, Lee HS (2009). Effect of caffeic acid on tumour necrosis factor-alpha-induced vascular inflammation in human umbilical vein endothelial cells. Biol Pharm Bull.

[22] Deuell KA, Callegari A, Giachelli CM, Rosenfeld ME, Scatena M (2012). RANKL enhances macrophage paracrine pro-calcific activity in high phosphate-treated smooth muscle cells: dependence on IL-6 and TNF-alpha. J Vasc Res.

[23] Hoge M, Amar S (2006). Role of interleukin-1 in bacterial athero-genesis. Drugs Today (Barc).

[24] Bhaskar V, Yin J, Mirza AM (2011). Monoclonal antibodies targeting IL-1 beta reduce biomarkers of atherosclerosis in vitro and inhibit atherosclerotic plaque formation in apolipoprotein E-deficient mice. Atherosclerosis.

[25] Chamberlain J, Francis S, Brookes Z (2009). Interleukin-1 regulates multiple atherogenic mechanisms in response to fat feeding. PLoS One.

[26] Jiang Y, Wang M, Huang K (2012). Oxidized low-density lipo-protein induces secretion of interleukin-1beta by macrophages via reactive oxygen species-dependent NLRP3 inflammasome activation. Biochem Biophys Res Commun.

[27] Sivapalaratnam S, Farrugia R, Nieuwdorp M (2011). Identification of candidate genes linking systemic inflammation to atherosclerosis; results of a human in vivo LPS infusion study. BMC Med Genomics.

[28] Navab M, Hough GP, Stevenson LW, Drinkwater DC, Laks H, Fogelman AM (1988). Monocyte migration into the subendo-thelial space of a coculture of adult human aortic endothelial and smooth muscle cells. J Clin Invest.

[29] Van Lenten BJ, Hama SY, de Beer FC (1995). Anti-inflammatory HDL becomes pro-inflammatory during the acute phase response. Loss of protective effect of HDL against LDL oxidation in aortic wall cell cocultures. J Clin Invest.

[30] Ishikawa K, Navab M, Leitinger N, Fogelman AM, Lusis AJ (1997). Induction of heme oxygenase-1 inhibits the monocyte transmigration induced by mildly oxidized LDL. J Clin Invest.

[31] Takaku M, Wada Y, Jinnouchi K (1999). An in vitro coculture model of transmigrant monocytes and foam cell formation. Arterioscler Thromb Vasc Biol.

[32] Zhang JC, Ruan Q, Paucz L, Fabry A, Binder BR, Wojta J (1999). Stimulation of tissue factor expression in human microvascular and macrovascular endothelial cells by cultured vascular smooth muscle cells in vitro. J Vasc Res.

[33] Rainger GE, Stone P, Morland CM, Nash GB (2001). A novel system for investigating the ability of smooth muscle cells and fibroblasts to regulate adhesion of flowing leukocytes to endothelial cells. J Immunol Methods.

[34] Chao CY, Lii CK, Tsai IT, Li CC, Liu KL, Tsai CW, Chen HW (2011). Andrographolide inhibits ICAM-1 expression and NF-κB activation in TNF-α-treated EA.hy926 cells. J Agric Food Chem.

[35] Wang YH, Yan ZQ, Qi YX (2010). Normal shear stress and vascular smooth muscle cells modulate migration of endothelial cells through histone deacetylase 6 activation and tubulin acetylation. Ann Biomed Eng.

[36] Evensen L, Micklem DR, Blois A (2009). Mural cell associated VEGF is required for organotypic vessel formation. PLoS One.

[37] Wang YH, Yan ZQ, Shen BR, Zhang L, Zhang P, Jiang ZL (2009). Vascular smooth muscle cells promote endothelial cell adhesion via microtubule dynamics and activation of paxillin and the extracellular signal-regulated kinase (ERK) pathway in a co-culture system. Eur J Cell Biol.

[38] Ruan LM, Cai W, Chen JZ, Duan JF (2010). Effects of losartan on expression of connexins at the early stage of atherosclerosis in rabbits. Int J Med Sci.

[39] Larson DM, Haudenschild CC, Beyer EC (1990). Gap junction messenger RNA expression by vascular wall cells. Circ Res.

[40] Navab M, Liao F, Hough GP (1991). Interaction of monocytes with cocultures of human aortic wall cells involves interleukins 1 and 6 with marked increases in connexin43 message. J Clin Invest.

[41] Leclercq A, Houard X, Loyau S (2007). Topology of protease activities reflects atherothrombotic plaque complexity. Atherosclerosis.

[42] Kuzuya M, Nakamura K, Sasaki T, Cheng XW, Itohara S, Iguchi A (2006). Effect of MMP-2 deficiency on atherosclerotic lesion formation in apoE-deficient mice. Arterioscler Thromb Vasc Biol.

[43] Lin R, Wang WR, Liu JT, Yang GD, Han CJ (2006). Protective effect of tanshinone IIA on human umbilical vein endothelial cell injured by hydrogen peroxide and its mechanism. J Ethnopharmacol.

[44] Zhang WL, Xiao Y, Liu JP (2011). Structure and remodeling behavior of drug-loaded high density lipoproteins and their atherosclerotic plaque targeting mechanism in foam cell model. Int J Pharm.

[45] Liu Z, Wang J, Huang E (2014). Tanshinone IIA suppresses cholesterol accumulation in human macrophages: role of haem oxygenase-1. J Lipid Res.

[46] Stumpf C, Fan Q, Hintermann C (2013). Anti-inflammatory effects of danshen on human vascular endothelial cells in culture. Am J Chin Med.

[47] Lee KC, Chang HH, Chung YH, Lee TY (2011). Andrographolide acts as an anti-inflammatory agent in LPS-stimulated RAW264.7 macrophages by inhibiting STAT3-mediated suppression of the NF-kappaB pathway. J Ethnopharmacol.

[48] Guo W, Liu W, Chen G (2012). Water-soluble androgra-pholide sulfonate exerts anti-sepsis action in mice through down-regulating p38 MAPK, STAT3 and NF-κB pathways. Int Immunopharmacol.

[49] Fang ZY, Lin R, Yuan BX, Yang GD, Liu Y, Zhang H (2008). Tanshinone IIA downregulates the CD40 expression and decreases MMP-2 activity on atherosclerosis induced by high fatty diet in rabbit. J Ethnopharmacol.

